# Neuronal and Neuroaxonal Damage Cerebrospinal Fluid Biomarkers in Autoimmune Encephalitis Associated or Not with the Presence of Tumor

**DOI:** 10.3390/biomedicines10061262

**Published:** 2022-05-28

**Authors:** Aigli G. Vakrakou, John S. Tzartos, Eleni Strataki, Fotini Boufidou, Eleni Dimou, Efstratios-Stylianos Pyrgelis, Vasilios C. Constantinides, George P. Paraskevas, Elisabeth Kapaki

**Affiliations:** 1 1st Department of Neurology, School of Medicine, National and Kapodistrian University of Athens, Eginition Hospital, 74, Vasilissis-Sofias-Avenue, 11528 Athens, Greece; avakrakou@gmail.com (A.G.V.); strataki.ln@gmail.com (E.S.); stratospyrg@yahoo.gr (E.-S.P.); vassilis.kon@hotmail.com (V.C.C.); geoprskvs44@gmail.com (G.P.P.); 2 2nd Department of Neurology, School of Medicine, National and Kapodistrian University of Athens, Attikon University Hospital, Rimini 1, 12462 Athens, Greece; jtzartos@gmail.com; 3 Tzartos NeuroDiagnostics, Neuroimmunology, Eslin Street 3, 11523 Athens, Greece; elenidimoukonst1@gmail.com; 4 1st Department of Neurology, Neurochemistry and Biological Markers Unit, School of Medicine, National and Kapodistrian University of Athens, Eginition Hospital, 74, Vass. Sophias Ave., 11528 Athens, Greece; fboufidou@med.uoa.gr

**Keywords:** autoimmune neurological syndrome, paraneoplastic neurological syndrome, non-paraneoplastic, autoimmune encephalitis, neurofilament light chain, total tau protein

## Abstract

The aim of this study was to evaluate the association of neuronal damage biomarkers (neurofilament light chain (NFL) and total tau protein (T-tau)) in the CSF of patients with autoimmune encephalitis (AE) with the presence of an underlying malignancy and to determine correlations with patient characteristics. The study comprised 21 patients with encephalitis associated with antibodies against intracellular (*n* = 11) and surface/synaptic antigens (extracellular, *n* = 10) and non-inflammatory disease controls (*n* = 10). Patients with AE associated with intracellular antigens had increased CSF-NFL (*p* = 0.003) but not T-tau levels compared to controls. When adjusted for age, CSF-NFL but not CSF-T-tau was higher in patients with encephalitis associated with intracellular antigens as compared to those with encephalitis associated with extracellular antigens (*p* = 0.032). Total tau and NFL levels were not significantly altered in patients with encephalitis associated with extracellular antigens compared to controls. NFL in the total cohort correlated with neurological signs of cerebellar dysfunction, peripheral neuropathy, presence of CV2 positivity, presence of an underlying tumor and a more detrimental clinical outcome. AE patients with abnormal MRI findings displayed higher NFL levels compared to those without, albeit with no statistical significance (*p* = 0.07). Using receiver operating characteristic curve analysis, CSF-NFL levels with a cut-off value of 969 pg/mL had a sensitivity and specificity of 100% and 76.19%, respectively, regarding the detection of underlying malignancies. Our findings suggest that neuronal integrity is preserved in autoimmune encephalitis associated with extracellular antigens and without the presence of tumor. However, highly increased NFL is observed in AE associated with intracellular antigens and presence of an underlying tumor. CSF-NFL could potentially be used as a diagnostic biomarker of underlying malignancies in the clinical setting of AE.

## 1. Introduction

Encephalitis is an inflammatory brain condition with many possible causes, and in 40–50% of cases the etiology cannot be established [1]. There are several types of encephalitis that are immune-mediated, including the classic paraneoplastic encephalitis syndromes, often associated with antibodies against intracellular neuronal proteins (onconeural antigens), and the encephalitis syndromes associated with antibodies against neuronal cell surface/synaptic proteins involved in neuronal signaling and synaptic plasticity, often referred to as “autoimmune encephalitis” [2]. While the paraneoplastic encephalitis syndromes are invariably cancer-related, the autoimmune encephalitis syndromes may occur in the presence or absence of cancer [2].

A definite diagnosis of paraneoplastic or autoimmune encephalitis can be difficult because of the broad spectrum of disease manifestations. Clinical presentation is regularly not limited to a well-defined syndrome and not specific enough to identify those patients with underlying malignancies [3]. Conventional neurological evaluation, standard diagnostic tests (e.g., MRI, CSF, EEG studies), and assessment of antibodies against intracellular and neuronal surface antigens can offer only limited diagnostic help in identifying tumor-associated neurological disorders [4]. The absence of autoantibodies does not exclude the possibility that a disorder is immune-mediated, and a positive test does not always imply an accurate diagnosis [3]. Typically, patients with malignancies cannot be identified before the onset of clinical symptoms related to the tumors. Nevertheless, timely detection of a malignancy is pivotal for improving the long-term prognosis; thus, additional biomarkers are needed to help establish prompt diagnoses.

Increased levels of neuroaxonal damage markers (neurofilament light chain (NFL) and total tau protein (T-tau)) have been found in patients with both paraneoplastic and non-paraneoplastic autoimmune syndromes [5,6], as well as in other neuroinflammatory conditions, such as myelin oligodendrocyte glycoprotein antibody disorders [7] and multiple sclerosis [8,9]. Moreover, no definite associations between NFL values, radiological abnormalities or other CSF data have been found [6].

To determine whether processes of neuronal and neuroaxonal injury associate with clinical course, disease severity and the presence of an underlying tumor in antibody-mediated encephalitis, we measured biomarkers of these processes in CSF from patients presenting with antibody-mediated encephalitis and from cognitively normal individuals.

## 2. Materials and Methods

### 2.1. Study Population

A total of 31 adults was included in the study. All participants were recruited during the years 2012–2020 and all fulfilled the criteria for autoimmune encephalitis as defined by Graus et al. [10]. Of these patients, 10 had no detectable known autoantibody, either in serum or CSF, and were thus excluded. The remaining 21 patients were subdivided into those with detectable antibodies to an intracellular antigen (*n* = 11, mean age: 69 ± 12) and those with detectable antibodies to an extracellular antigen (*n* = 10, mean age: 46.9 ± 21). Ten healthy controls (mean age: 71.7 ± 4) undergoing minor surgery (such as hernia repair or knee joint surgery under spinal anesthesia) were included for comparisons. The control group presented no cognitive complaints and no neurological, psychiatric or other major diseases. They all had normal cognitive function as assessed by a semi-structured interview based on the Mini-Mental State Examination (MMSE) and within-normal-limit scores for neuropsychological testing based on the Hamilton Depression Rating Scale (HAM-D) prior to operation. Prior history of underlying malignancy was negative.

### 2.2. Diagnostic Evaluation

Demographic and clinical data at initial presentation and at follow-up were collected in all cases retrospectively. The presence of underlying malignancies was also reported. All patients underwent a complete physical and neurological examination, evaluating focal and peripheral symptoms/signs, autonomic dysfunction, psychiatric symptoms, cognitive impairment and movement disorders. A battery of neuropsychological tests was performed in all patients which included: (a) the Mini-Mental State Examination (MMSE); (b) the Frontal Assessment Battery (FAB); (c) the 5-word immediate and delayed recall (5WT); and (d) the 15-point spontaneous and copy CLOX drawing (CLOX1 and 2, respectively) tests. Neuropsychiatric tests included the 15-item Geriatric Depression Scale (GDS).

Laboratory investigations included electroencephalogram (EEG); electromyography (EMG); 3 or 1.5 Tesla MRI brain (some earlier MRIs were not available due to the retrospective nature of the study); abdomen, chest and pelvis CT; a standard commercially available panel of paraneoplastic and autoimmune antibodies (NMDAR, AMPAR, Caspr2, LGI, GABAb, GlyR, Yo, Ri, Ma2, CV2/CRMP5, Hu, Zic4, Tr, GAD and amphiphysin) in the serum and/or CSF; CSF analysis (number of cells, protein content and oligoclonal bands); and Positron Emission Tomography and Computed Tomography (PET-CT) if necessary.

### 2.3. CSF Measurements

Lumbar puncture was performed at 10–11 am, after overnight fasting, at the L3–L5 interspace, according to recently proposed recommendations on standardized operating procedures for CSF biomarkers [11]. In brief, four polypropylene tubes were used for CSF collection. The initial tube (2 mL) was used for routine cytology and biochemistry. The next tube (4 mL) was used for syphilis serology or any other determination suggested by the clinical presentation. The last tube of 5 mL was immediately centrifuged, aliquoted in polypropylene tubes (750 μL each) and, finally, stored at −80 °C until analysis. Aliquots were thawed only once, just before analysis.

The CSF levels of total tau protein and NFL were measured in duplicate by enzyme-linked immunosorbent assays (Human total tau ELISA kit, Neuroimmune, Germany, and NF-light^®^ Elisa, UmanDiagnostics AB, Sweden) according to the manufacturers’ instructions. The concentration of total tau and NFL (picograms per milliliter) were calculated using standard curves. The detection limit of NF-light^®^ Elisa is 33 pg/mL. All CSF analyses were performed in samples collected during disease presentation (upon disease exacerbation).

### 2.4. Statistical Analysis

Data were first tested for normal distribution. Due to non-normal distribution, the Mann–Whitney rank-sum test was used to compare between groups. A two tailed *p*-value < 0.05 was considered statistically significant. Group analysis of patients involved various clinical/serological/radiological parameters as reported in the patients’ characteristics. To control for multiple comparisons, a Holm–Sidak test was performed. Subsequently, to test the sensitivity and specificity of the tested biomarkers, receiver operating characteristic (ROC) curves were applied. Biomarker levels were compared between groups of interest using a univariate analysis of covariance (ANCOVA), with age added as a variable covariate. Correlations were assessed by Spearman’s correlation coefficient. Statistical analysis was performed using GraphPad Prism-9.

## 3. Results

### 3.1. Patient Characteristics

Of the 21 patients with encephalitis included in the study, 11 had antibodies against intracellular antigens (Yo, *n* = 2; Ma2, *n* = 1; CV2/CRMP5, *n* = 1; CV2/CRMP5 and Yo, *n* = 1; CV2/CRMP5 and Hu, *n* = 2; Zic4, *n* = 1; GAD, *n* = 2; and amphiphysin, *n* = 1) and 10 patients had antibodies against extracellular antigens (NMDAR, *n* = 4; Caspr2, *n* = 2; GABAbR, *n* = 1; GlyR, *n* = 3). Malignancy was detected in 10 patients. Of these patients, eight had antibodies against intracellular antigens and two had antigens against neuronal cell surface antigens. Demographic/clinical data and paraclinical data of the patients are reported in Table 1 and Supplementary Table S1.

### 3.2. CSF Brain Damage Markers

**Figure 1:**
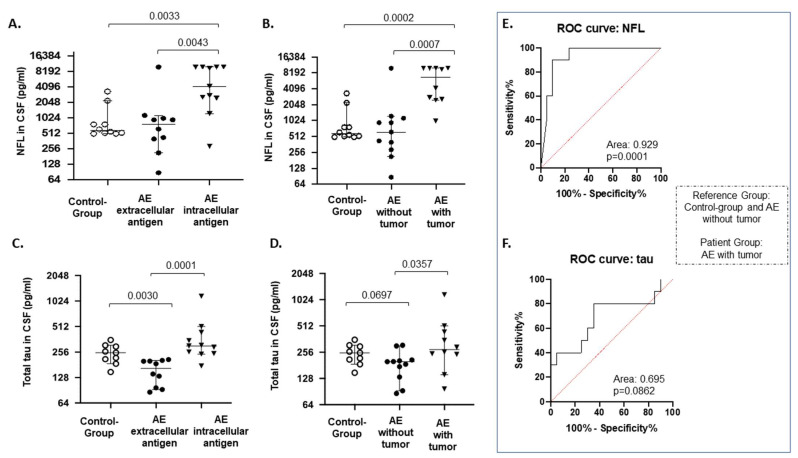
Biomarkers of neuronal and neuroaxonal injury at disease exacerbation. (**A**,**B**) Cerebrospinal fluid (CSF) NFL levels and (**C**,**D**) total tau levels were compared between the control group (*n* = 10) and patients with autoimmune encephalitis (AE). In panels A and C, each group of patients was subdivided into those associated with the presence of autoantibodies against extracellular antigens and those associated with the presence of autoantibodies against intracellular antigens. In panels B and D, each group of patients was subdivided into the those associated with the presence of an underlying tumor and those not associated with the presence of tumor. (**E**,**F**) Receiver operating characteristic curve (ROC) analysis displaying the trade-off between sensitivity (true-positive rate) and (100% specificity) (false-positive rate). ROC curves tested the ability of each biomarker to discriminate the reference group (the control group and patients with AE without tumors) from the group of interest (patients with AE and tumors). For NFL: area under the curve = 0.9286, standard error = 0.04694 and 95% confidence interval: 0.8366 to 1.000; *p*-value = 0.0001. This analysis indicated that the best cut-off for CSF-NFL was >969 pg/mL, with the corresponding sensitivity and specificity to predict the presence of tumor set at 100% and 76.19%, respectively (**E**). ROC curve statistics for total tau were not statistically significant: area under the curve = 0.6950, *p* = 0.0862 (**F**). The horizontal lines in the dots represent the median. The vertical lines indicate the 95% CI, confidence interval. The *p*-values were obtained via unpaired *t*-test analysis using the Mann–Whitney test. *p*-values are indicated in the figure, and statistically significant comparisons were considered as those with *p* < 0.05. CSF = cerebrospinal fluid; AE = autoimmune encephalitis; NFL = neurofilament light chain; ROC = receiver operating characteristic curve.

Of the total study cohort, the highest CSF-NFL levels were found in patients with antibodies against CV2 alone (*n* = 1; >10,000 pg/mL), both CV2 and Yo (*n* = 1; >10,000 pg/mL) and both CV2 and Hu (*n* = 2; 9459 and >10,000 pg/mL, respectively), while the highest CSF-tau levels were found in patients with antibodies against Ma2/Ta (*n* = 1; 510.9 pg/mL), CV2 and Hu (*n* = 2; 256.7 and 1162.8 pg/mL, respectively,) and Zic4 (*n* = 1; 434 pg/mL,) (Table 2). When the patients were grouped based on the localization of antigens, CSF-NFL levels were higher in patients with antibodies against intracellular antigens (median: 5728 pg/mL, range: 288–10,000) compared to those with antibodies against cell surface antigens (median: 1575 pg/mL, range: 87–10,000; *p* = 0.0043) and the controls (median: 1029 pg/mL, range: 502–2844; *p* = 0.0033) (Figure 1A). NFL levels were further compared among patients with antibodies against intracellular antigens and without using a univariate analysis of covariance (ANCOVA), with age added as a variable covariate. The adjusted *p*-value for NFL was still significantly increased in those with the presence of intracellular antigens (adjusted *p*-value = 0.032).

CSF-tau was significantly higher in patients with antibodies against intracellular antigens (median: 388.7, range: 175–1163) compared to those with antibodies against cell surface antigens (median: 154.9, range: 86–209.1; *p* = 0.0001) (Figure 1C). The adjusted *p*-value when correcting for age did not reach statistical significance (adjusted *p*-value = 0.095). Moreover, CSF-tau was lower in the patients with antibodies to cell surface antigens than in the controls (median: 248.4, range: 149–354; *p* = 0.003; adjusted for age, *p*-value = 0.092) (Figure 1C). In our cohort, age was an important co-factor mainly affecting total tau levels (Supplementary Table S2).

### 3.3. Correlation of CSF Brain Damage Markers with Various Clinical and Radiological Parameters

In the total patient cohort, CSF-NFL levels were found to correlate with the following parameters (Spearman’s R): age at disease onset (r = 0.45, *p* = 0.04), CRMP5/CV2 positivity (r = 0.58, *p* = 0.005), dysmetria (r = 0.4367, *p* = 0.05), cerebellar ataxia (r = 0.46, *p* = 0.04), balance disorder (r = 0.48, *p* = 0.03), respiratory failure (r = 0.58, *p* = 0.005), peripheral neuropathy (r = 0.65, *p* = 0.001), tumor presence (r = 0.71, *p* = 0.0003) and death (r = 0.6, *p* = 0.017). Moreover, CSF-NFL levels were found to correlate with specific neuropsychological variables; significant correlations were observed among CSF-NFL with decreasing MMSE values (Spearman’s R = −0.5122, *p* = 0.0250), FAB (Spearman’s R = −0.4841, *p* = 0.0418) and Clox2 (Spearman’s R = −0.6677, *p* = 0.0044)—a pattern of cognitive disturbances indicative of loss of visuomotor functionalities. Moreover, CSF-NFL showed a negative correlation with levels of total IgG in the serum (Spearman’s R = −0.6439, *p* = 0.0114). Finally, a moderate correlation was found between CSF-NFL and CSF-tau levels (Spearman’s R = 0.4793 *p* = 0.0279).

CSF-T-tau levels in the total patient cohort correlated with age at disease onset (r = 0.71, *p* = 0.0003), GAD positivity (r = 0.47, *p* = 0.03), NMDAR positivity (r= −0.56, *p* = 0.008), bradykinesia (r = 0.52, *p* = 0.02), dysdiadochokinesia (r = 0.47, *p* = 0.03), weight loss (r = 0.48, *p* = 0.03), peripheral neuropathy (r = 0.46, *p* = 0.04), tumor presence (r = 0.47, *p* = 0.03), partial recovery (regarding disease evolution) (r = −0.53, *p* = 0.04) and death (r = 0.66, *p* = 0.008) (independent correlations, not correction for multiple parameters).

### 3.4. Brain MRI

At disease presentation, during acute phase, 8 out of 21 patients presented brain MRI abnormalities.

**Figure 2:**
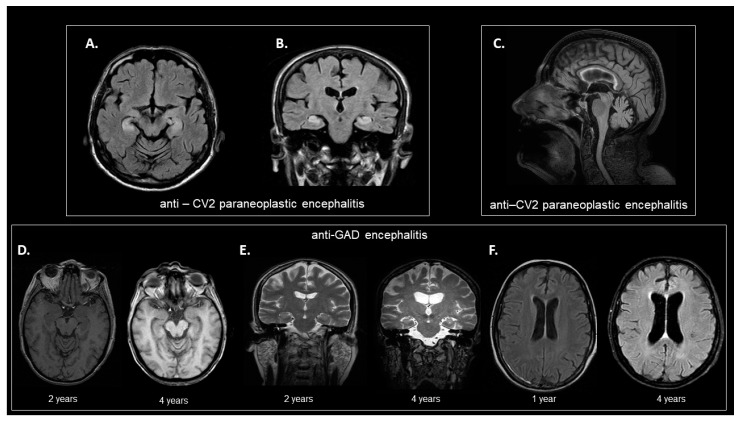
Representative MRI findings of our study cohort. (**A**,**B**) Brain MRI from a 72-year-old male patient with a diagnosis of anti-CV2 paraneoplastic encephalitis. Axial (**D**) and coronal (**E**) T2-weighted FLAIR images (at 1.5T). T2-weighted FLAIR signal hyperintensities can be seen in the hippocampi and amygdalas bilaterally. (**C**) Example of brain MRI from an 82-year-old female patient with a diagnosis of anti-CV2 paraneoplastic encephalitis. Sagittal FLAIR sequence (at 3T) showing signs of cerebellar vermal atrophy. The atrophy of the anterior lobe of cerebellar vermis (with dilatation of the primary fissure) is disproportionate to posterior lobe atrophy of cerebellar vermis and relative sparing of other fissures. (**D**,**F**) Example of brain MRI from a 58-year-old female patient with a diagnosis of anti-GAD autoimmune encephalitis demonstrating dilatation of the ventricular system and cortical and hippocampal atrophy over the course of the disease. (**D**) Left: Axial T1-weighted 3D spoiled gradient echo sequence (at 1.5T); image acquired at 2 years after disease onset. Right: axial T1-weighted Turbo field echo sequence (at 3T); image acquired at 4 years after disease onset. (**E**) Left: Coronal T2-weighted fast spin echo sequence (at 1.5T); image acquired at 2 years after disease onset. Right: Coronal T2-weighted 3D sequence (at 3T); image acquired at 4 years after disease onset. (**F**) Left: Axial T2-weighted FLAIR sequence (at 1.5T); image acquired at 1 year after disease onset. Right: Axial T2-weighted FLAIR sequence (at 3T); image acquired at 4 years after disease onset. Note the enlargement of the temporal horns of the lateral ventricles (Scheltens 2 bilaterally) in panels A and B, and signs of cortical atrophy (GCA 1) four years after disease onset, especially in panels B and C. The rate of ventricular enlargement and cortical atrophy (in particular, as depicted by temporal horn enlargement) is particularly high for a 3-year period. MRI = magnetic resonance imaging; FLAIR = fluid-attenuated inversion recovery; GAD = glutamic acid decarboxylase; CV2/CRMP5 = collapsin response mediator protein; GCA= cortical atrophy.

Only one patient displayed typical findings suggestive of limbic encephalitis with a FLAIR-intense signal in the limbic system (Figure 2A,B) and another increased T2 signal in the basal ganglia (anti-CV2 and Yo encephalitis), whereas others displayed more atypical findings, such as cerebellar vermal atrophy (anti-CV2 encephalitis, Figure 2C), cortical and hippocampal atrophy (anti-GAD encephalitis, Figure 2D–F), generalized atrophy (anti-MA2, *n* = 1; anti-Yo, *n* = 1; anti-caspr−2, *n* = 1), hippocampal asymmetry (anti-NMDAR, *n* = 1). CSF-T-tau levels were not statistically different among patients with normal and abnormal brain MRI findings (including typical and atypical imaging findings). Patients with abnormal MRI findings displayed high NFL levels (mean level: 5947 pg/mL, SD: 4344) compared to those without (mean level: 2399 pg/mL, SD: 3466). However, this comparison did not reach statistical significance (*p* = 0.07).

### 3.5. Presence of Tumor

When the patients were grouped according to presence or absence of tumor, CSF-NFL levels were significantly increased in the patients with tumors (median: 6248, range: 1003–10,000) compared to those without (median: 1480, range: 87–10,000; *p* = 0.0007) and the controls (median: 1029, range: 502–3346; *p* = 0.0002) (Figure 1B). The adjusted *p*-value for NFL was still significantly increased in those with the presence of tumor (adjusted *p*-value = 0.01). CSF-tau levels were also found to be increased in patients with encephalitis associated with tumors (median: 373, range: 97–1163) compared to those without (median: 190.3, range: 86–221; *p* = 0.04) (Figure 1D). Nevertheless, the adjusted *p*-value was not significant when this biomarker was corrected for age. The presence of tumor was associated more strongly with CSF-NFL levels and cerebellar ataxia (Supplementary Table S3).

### 3.6. ROC Curve Analysis

ROC curve analysis was performed to investigate the possible diagnostic utility of CSF-NFL and CSF-T-tau levels for the detection of underlying tumors. Only CSF-NFL showed an acceptable diagnostic performance; we found that CSF-NFL levels at a cut-off of 969 pg/mL could differentiate the patients with encephalitis associated with tumors from those without tumors and the controls, with an area under the ROC curve of 0.93, excellent sensitivity (100%) and moderate specificity (76%) (Figure 1E,F).

## 4. Discussion

### 4.1. Major Findings

In the present study, we measured NFL and T-tau levels in the CSF of patients with neurological syndromes associated with autoantibodies to cell surface and intracellular/synaptic antigens. We found that patients with encephalitis associated with intracellular antigens had increased CSF-NFL but not T-tau levels as compared to controls. Furthermore, CSF-NFL and CSF-T-tau were higher in cases of encephalitis associated with intracellular antigens compared to those associated with cell surface/synaptic antigens. After controlling for age, only NFL levels differed between cell surface/synaptic and intracellular antigens. Finally, we found that CSF-NFL levels at a cut-off value of 969 pg/mL have a potential diagnostic utility in detecting underlying malignancies. Longitudinal analyses and validation of NFL as a candidate biomarker for paraneoplastic encephalitis in larger multicenter cohorts have to be carried out for its establishment in clinical practice.

### 4.2. Total Tau Levels in AE Associated with Extracellular Antigens—Minor Changes

**Figure 3:**
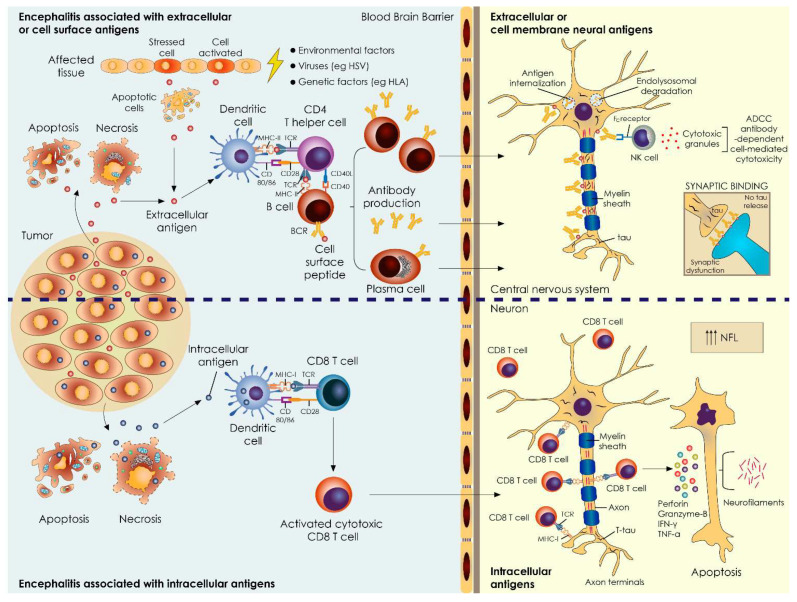
General principles of immune-pathogenesis of paraneoplastic and non-paraneoplastic autoimmune encephalitic syndromes and the role of neuronal and neuroaxonal damage biomarkers. The presence of tumor is more commonly found in autoimmune encephalitis associated with intracellular antigens (**lower panel**). Autoantigens expressed or released (either after apoptotic or necrotic cell death) by tumor cells are thought to initiate an autoimmune response especially after being captured by antigen-presenting cells (APCs) (e.g., dendritic cells) in peripheral lymphoid tissue (**lower panel**). In the absence of a tumor, the underlying pathology is obscure and the most common triggering factors are thought to be a viral infection or genetic/environmental factors affecting tissue homeostasis (**upper panel**). Extracellular antigens are processed and presented by APCs to CD4+ helper T cells in the context of MHC II molecules and fully activate B cells. B cells differentiate in antibody-producing plasma cells. B cells, plasma cells and IgG autoantibodies enter the central nervous system through a leaky blood–brain barrier. IgG autoantibodies have the potential to cause synaptic autoimmune disorders directly through antigen internalization, complement activation, antibody-dependent cell cytotoxicity (ADCC) and receptor blocking (**upper panel**). Intracellular antigens processed by APCs, in the context of MHC class I molecules and in the presence of a pro-inflammatory cytokine milieu, favor the activation of cytotoxic CD8+ effector T cells. Cytotoxic CD8 T cells invade the CNS to exert cellular neuron-directed autoimmunity via the release of perforin and granzyme B, leading to the apoptosis of the target cells (**lower panel**). CSF-NFL levels are elevated in cases of encephalitis associated with the presence of an underlying tumor, where cytotoxic CD8 T causes more destructive effects in the affected tissue (**lower panel**). CSF-NFL and tau are not elevated in patients with encephalitis associated with extracellular antigens, possibly reflecting less destructive effects of autoantibody-mediated neuroaxonal injury (e.g., dysfunction of neuronal synapsis, indicative of distinct antibody-mediated effects) (**upper panel**). APC = antigen-presenting cells; HLA = human leukocyte antigen; MHC = major histocompatibility complex; ADCC = antibody-dependent cell cytotoxicity; NFL = neurofilament, HSV = herpes simplex virus; BCR = B cell receptor, TCR = T cell receptor.

Antibodies in AE are mostly directed against neuronal surface antigens. By binding to excitatory transmitter receptors (NMDA, AMPA), inhibitory transmitter receptors (GABAb, GABAa, glycine), ion channel subunits and cell adhesion molecules (CASPR2, IgLON5) or soluble synaptic proteins (LGI1), they cause initially reversible neuronal dysfunction [12]. Autoantibody formation can be triggered by viral or tumor exposition, but the mechanisms are unknown in most cases. Since the role of these antibodies is directly pathogenic, patients with AE often respond favorably to treatments aimed at removing antibodies or antibody-producing cells and have a good chance of substantial recovery despite the severity of their symptoms [13]. Interestingly, we found that total tau levels were slightly decreased in patients with encephalitis associated with extracellular antigens. Total tau represents a marker of neuronal damage and it is primarily found in the axons of mature neurons, though it is also distributed in cell bodies, dendrites and dendritic spines [14,15]. Tau protein, apart from its known functions in stabilizing microtubule assembly and its involvement in axonal maturation and transport, also exhibits other delegate roles, such as involvement in synaptic plasticity, regulation of NMDA/AMPA receptors and interaction with membrane proteins controlling vesicle release [16,17]. The main pathogenetic mechanisms involved in encephalitis associated with extracellular antigens involve primarily functional aberrancies, such as antibody-mediated internalization of cell surface receptors, modulation of cell surface density and the mobility of the involved antigens or antibody-mediated blocking of receptors or stimulation thereof [18]. Therefore, we hypothesize that the trend towards lower total tau levels may reflect the maintenance of neuronal integrity along with synaptic dysfunction (Figure 3). Whether antibody-mediated functional effects induce reductions in tau through alterations to MAPT (microtubule-associated protein tau gene encoding tau) transcription, neurotransmission (tau translocation or compartmentalization) or synaptic vesicle mobility/release remains to be elucidated. Our findings are consistent with a recent report suggesting that neuronal integrity biomarkers in CSF are maintained in antibody-mediated encephalitis, despite neuroaxonal compromise [19]. Another study found that T-tau was only elevated in the CSF of NMDAR encephalitis patients with temporal FLAIR signals in MRIs and patients who develop hippocampal sclerosis [20].

### 4.3. NFL Levels in AE Associated with Intracellular/Synaptic Antigens Correlate with Disease Severity and Coexistence of Tumors

Intracellular neuronal antigens, i.e., Hu, Ri, Yo and Ma2, are known to be targeted by autoantibodies in paraneoplastic neurological syndromes (PNSs) and these autoantibodies serve as valuable biomarkers indicating the presence of underlying tumors [13]. Primarily autoreactive cytotoxic T cells and not observed humoral immune responses are considered the major mediators of neuronal damage. Target antigens (onconeural) of the autoreactive process are immunogenic neoantigens ectopically produced by tumor cells and normally expressed by neurons [2]. Among antibodies targeting intracellular/synaptic antigens, GAD antibodies are only rarely associated with tumors [21]. PNSs tend to affect older individuals [22] and every level of the nervous system can be affected [13]; indeed, the patients in the study presented with a broad spectrum of clinical symptoms/signs, including cognitive decline, memory deficits and movement disorders. In contrast to AEs, patients with paraneoplastic syndromes rarely respond to treatment aimed at removing antibodies or antibody-producing cells [13]. In the majority of cases, symptoms have an acute to subacute onset, and in more than half of patients the neurological syndrome develops before the cancer diagnosis is known. Cancer types that associate with antibodies against intracellular antigens are small cell lung, gynecological, breast, thymoma, Hodgkin’s lymphoma, and testicular cancers [13]. Of the patients with a cancer diagnosis included in the study, two patients were diagnosed with ovarian cancer; one patient with nasopharynx, mediastinal, endometrium, and prostate cancer; and four patients with lung cancer (both small cell and non-small cell).

Neuropathological studies of patients with PNSs have mainly evaluated the immunological aspects of disease pathogenesis, with an emphasis on characterizations of inflammatory infiltrates (perivascular CD4 T cells and B cells, parenchymal CD8 T cells and macrophages), gliosis, microglia activation, neuronal loss and neuronophagia. Nevertheless, a detailed investigation of tissue markers of neurodegeneration has not been performed. NFL is mainly expressed in high-caliber myelinated axons within the central nervous systems, especially in subcortical regions, and is considered to indicate neuroaxonal damage and degeneration, irrespective of cause [23]. Herein, we found that mainly CSF-NFL and to lesser extent total tau levels were elevated in patients with antibodies against intracellular antigens compared to those with antibodies against neuronal cell surface antigens (Figure 3). In contrast to the study of Constantinescu et al. [5] reporting that CSF-NFL levels are not diagnostic for malignancy after age-adjustment, we found a statistical correlation between CSF-NFL levels and presence of tumor after correcting for age. This discrepancy could be attributed to different study cohorts and methods of analysis. Using ROC curve analysis, we showed that CSF-NFL levels at a cut-off value of 969 pg/mL had an excellent sensitivity and moderate specificity of 100% and 76.19%, respectively, in the diagnosis of an underlying malignancy [24]. Recently, it has been shown that NFL in the serum and CSF do not associate with the MRI/CSF inflammatory profile of antibody-mediated encephalitis, though it seems to better facilitate longitudinal monitoring of disease activity [25]. It remains unclear whether the increased NFL in CSF is caused directly by inflammatory cell-mediated damage or by active secretion as result of remodeling processes after brain tissue damage [26].

### 4.4. Limitations

This study has several limitations, including those associated with the retrospective sampling of CSF from a relatively small cohort of patients assessed at one academic medical center and the limited numbers of sequential MRIs for some patients (especially earlier ones). Nevertheless, the extended presentation of clinical information is one of the major powers of the present study. Prospective evaluation of greater numbers of patients with autoimmune encephalitis is required to validate the findings. The pathomechanisms of autoimmune encephalitis are relevantly different for each antibody and thus larger groups with the same antibody with/without tumors are needed to finally answer our research questions. Of note, we should keep in mind that the only way to diagnose autoimmune encephalitis with certainty is by pathology. Based on clinical symptoms alone, it is extremely challenging to identify the differences in CSF markers and the specificity of lesions, if existent, on imaging.

## 5. Conclusions

The patterns of CSF biomarkers of neuronal and neuroaxonal damage in autoimmune neurological syndromes associated with antibodies against extracellular antigens showed distinct features compared to those associated with antibodies against intracellular antigens—a finding that implicates a difference in pathogenetic mechanisms between them. Our findings suggest that CSF-NFL could potentially be used as a diagnostic biomarker of encephalitis with underlying malignancies. In the CSF of antibody-mediated encephalitis, the relative maintenance of biomarkers of synaptic and/or neuroaxonal integrity (CSF-tau and NFL), possibly reflects distinct antibody-mediated effects. Future studies with larger cohorts are warranted to validate the benefit of NFL and total tau measures in clinical practice.

## Figures and Tables

**Table 1:** Demographic and clinical/paraclinical data of the patients included in the study.

Demographics	
Patients, number	21
Age at disease onset, median (years)	67
Female, *n* (%)	12 (57.1)
**Antibody status, *n* (%)**	
**Intracellular/synaptic antigens, *n* = 11**	
Yo	2 (9.52)
Ma2	1 (4.76)
CV2/CRMP5	1 (4.76)
CV2/CRMP5 and Yo	1 (4.76)
CV2/CRMP5 and Hu	2 (9.52)
Zic4	1 (4.76)
GAD	2 (9.52)
Amphiphysin	1 (4.76)
**Neuronal cell surface antigens, *n* = 10**	
NMDAR	4 (19.05)
Caspr2	2 (9.52)
GABAb-R	1 (4.76)
**Antibody detection, *n* (%)**	
Serum only	19 (90.48)
CSF only	2 (9.52)
Both serum and CSF	8 (38.1)
**Clinical presentation/symptoms, *n* (%)**	
Cognitive decline	14 (66.67)
Memory deficits	13 (61.9)
Spatial disorientation	9 (42.86)
Altered level of consciousness	2 (9.52)
Change in behaviour	8 (38.1)
Psychiatric symptoms	12 (57.14)
Dysarthria	4 (19.05)
Seizures	7 (33.33)
Peripheral nerve involvement (sensory and/or motor neuropathy)	4 (19.05)
**Selected clinical signs, *n* (%)**	
Autonomic dysfunction	6 (28.57)
Parkinsonism	7 (33.33)
Dystonia	4 (19.05)
Cerebellar ataxia	9 (42.86)
Orofacial and limb dyskinesia	2 (9.52)
Ocular flatter	1 (4.76)
Opsoclonus	1 (4.76)
Acquired hyperekplexia	2 (9.52)
**Tumor association**	
**Tumor type,** total *n* = 10, *n* (% of total tumors)	
Nasopharynx cancer	1 (10)
Mediastinal	1 (10)
Lung cancer	4 (40)
Ovarian	2 (20)
Endometrium	1 (10)
Prostate	1 (10)
**Disease outcome, *n* (%)**	
Significant improvement	5 (23.81)
Partial improvement	7 (33.33)
Stable	2 (9.52)
Worsening of symptoms	3 (14.29)
Death	4 (19.05)
**MRI findings**	
**Brain MRI scans**	
Abnormal findings related to encephalitis, *n* (%)	5 (23.81)
Significant atrophic changes, disproportionate to age, *n* (%)	2 (9.52)
Basal ganglia involvement, *n* (%)	1 (4.76)
Hippocampal involvement, *n* (%)	2 (9.52)
Gadolinium enhancement, *n* (%)	0
**EEG studies**	
Normal, *n* (%)	10 (47.62)
Focal showing, *n* (%)	7 (33.33)
Generalized showing, *n* (%)	4 (19.05)
Classic epileptic from discharge, *n* (%)	5 (23.81)
Delta brush, *n* (%)	2 (9.52)
**Treatment modalities**	
Steroids (intravenous administration), *n* (%)	14 (66.67)
Plasmapheresis, *n* (%)	6 (28.57)
IVIG, *n* (%)	2 (9.52)
Cyclophosphamide, *n* (%)	2 (9.52)
Anti-CD20 therapy, *n* (%)	1 (4.76)

Demographic and clinical features of 21 patients with autoimmune encephalitis syndromes. Psychiatric symptoms = any of agitation/anxiety/depression/hallucinations (visual/auditory)/psychosis/delirium. Seizures = any of faciobrachial dystonic spasms, subtle focal tonic–clonic, temporal lobe seizures. Autonomic dysfunction = any of hyperhidrosis, tachycardia/arrhythmia, urinary and or gastrointestinal dysfunction, Adie pupil, sexual dysfunction, blood pressure abnormalities. Parkinsonism = any of bradykinesia, tremor, rigidity. Abbreviations; Anti-CV2/CRMP5 = collapsin response mediator protein 5; Ma2 = membrane active protein 2; CASPR2 = contactin-associated protein-like 2; CRMP5 = collapsin response mediator protein 5; FLAIR = fluid-attenuated inversion recovery; GABABR = gamma-aminobutyric acid receptor B; GAD = glutamic acid decarboxylase; Zic4 = Zic family member 4; GlyR = glycine receptor; IVIG = intravenous immune globulin; EEG = electroencephalogram; MRI = magnetic resonance imaging.

**Table 2:** CSF NFL and CSF total tau levels (pg/mL) in patients with various encephalitis syndromes associated with antibodies directed against either extracellular or intracellular/synaptic antigens.

CSF Biomarkers in Antibody-Associated Encephalitis		
	NFL Mean (SD)	Total Tau Mean (SD)
Controls, *n* = 10	1029 (965)	248 (64)
**Extracellular/synaptic antigens**		
Abs directed against:		
NMDA-R, *n* = 4	2826 (4800)	128 (46)
CASPR2, *n* = 2	1036	167
Gly-R, *n* = 3	585 (303)	165 (47)
GABAbR, *n* = 1	620	209
**Intracellular/synaptic antigens**		
Yo, *n* = 2	2679	295
Ma2, *n* = 1	4187	511
CV2/CRMP5, *n* = 1	>10,000	242
Yo, *n* = 2	2679	319
CV2/CRMP5 and Hu, *n* = 2	9730	710
CV2/CRMP5 and Yo, *n* = 1	>10,000	246
Zic4, *n* = 1	2477	435
GAD, *n* = 2	5144	304
amphiphysin, *n* = 1	1241	175

Abbreviations; Anti-CV2/CRMP5 = collapsin response mediator protein 5; Ma2 = membrane active protein 2; CASPR2 = contactin-associated protein-like 2; CRMP5 = collapsin response mediator protein 5, GABABR = gamma-aminobutyric acid receptor B; GAD = glutamic acid decarboxylase; Zic4 = Zic family member 4; GlyR = glycine receptor.

## Data Availability

All data are reported within the article and are available (anonymized) by request from the qualified investigators.

## Supplementary Materials

The following supporting information can be downloaded at: https://www.mdpi.com/article/10.3390/biomedicines10061262/s1, Table S1: More detailed demographic and clinical/paraclinical data for the patients included in the study; Table S2: Significant differences in various parameters tested among patients with autoimmune encephalitis with autoantibodies against extracellular versus intracellular antigens (without correction for multiple comparisons); Table S3: Significant differences in various parameters tested among patients with autoimmune encephalitis associated or not with the presence of an underlying tumor (without correction for multiple comparisons).
