# Supplementary material for: Neuronal and Neuroaxonal Damage Cerebrospinal Fluid Biomarkers in Autoimmune Encephalitis Associated or Not with the Presence of Tumor
Source: Biomedicines. 2022 May 28;10(6):1262. doi: 10.3390/biomedicines10061262 (PMC9220160; doi:10.3390/biomedicines10061262)
Supplement: Supplementary file 1 [file biomedicines-10-01262-s001.zip › Revised Supplementary Tables with minor change-1.pdf]

Supplementary Table 1– Summary of demographic, clinical, laboratory and radiological features in 21 patients with autoimmune and paraneoplastic encephalitis.

Patient #/sex/age at onset (years)	Classification	Antibody Type	Serum Detection	CSF Detection	History of autoimmunity	Clinical Presentation & phenotypical characteristics	Neurological and Physical Examination findings	Cognitive impairment	Brain MRI findings	Presence of tumour	EEG findings	EMG findings
1/F/31	Autoimmune encephalitis/ extracellular antigen	NMDAR	Yes	Yes	No	Delirium, psychosis and memory deficits of acute onset following ovarian cyst removal surgery	Confusion, disorientation, irritability, emotional lability, nystagmus, presence of thought and perception disorders, cerebellar ataxia, Orthostatic Hypotension	Yes	Normal	Yes; ovarian cystic teratoma	Focal delta waves left frontal-temporal areas and general slowing	Normal
2/F/24	Autoimmune encephalitis/ extracellular antigen	NMDAR	N/A	N/A	Yes; Previously diagnosed NMDAR encephalitis	Fatigue, headache, fever followed by epileptic tonic-clonic seizure, personality changes, memory deficits, communication disorder, headache and nausea possibly in the context of a NMDA encephalitis	Confusion, disorientation, irritability, oppositionality, emotional lability, dysarthria, nystagmus, brisk tendon reflexes, dyskinesia; Autonomic dysfunction features:	Yes	Hippocampal asymmetry	No	Generalized slowing, mainly in left temporal areas with sharp-like wave morphology	Normal

						relapse of subacute onset	Arrythmias, gastrointestinal dysfunction, orthostatic hypotension, pre-syncope				
3/M/61	Autoimmune encephalitis/ extracellular antigen	Caspr-2	Yes	Yes	No	Dizziness, loss of consciousness event possibly of epileptic aetiology with subsequent mild confusion, irritability and short – term memory impairment of acute onset	Confusion, disorientation, irritability, memory deficits, brisk tendon reflexes, extensor plantar reflex, numbness, Pre-syncope	Yes	White matter hyperintensities on T2WI/FLAIR (Fazekas1)	No	Epileptiform activity with sharp waves in left temporal areas	Normal
4/M/64	Autoimmune encephalitis/ extracellular antigen	Caspr-2	Yes	Yes	No	Late onset epilepsy with focal seizures 3 years prior to admission followed by cognitive impairment – memory and behaviour disorder. Symptoms precipitated during the last 6months	Irritability, oppositionality, emotional lability, memory and concentration deficits, mild dysarthria, mild muscle atrophies, hypertonia, brisk tendon reflexes, numbness	Yes	White matter hyperintensities (Fazekas 2) Diffuse mild cortical atrophy Sheltens 1-2	No	Slowing - delta waves mainly left temporal and sharp waves mainly in right temporal-parietal areas	

5/M/68	Autoimmune encephalitis/ extracellular antigen	GlyR	Yes	Yes	No	Mandibular rigidity that rapidly progressed to lockjaw, hyperekplexia, involuntary movements of the left hemiface and left upper extremity of acute to subacute onset	Hemifacial tactile sensitivity mainly in V2 – V3 branches of the trigeminal nerve, masseter muscle hypertonia, end-point tremor, hyperreflexia and hyperekplexia	No	Normal Fazekas 1 Mild symmetric frontoparietal atrophy	No	Normal	Normal
6/F/67	Autoimmune encephalitis/ extracellular antigen	GlyR, Recoverin	Yes	No	Yes; Possible Hashimoto's thyroiditis	Drug-resistant psychiatric symptoms including delusions of persecution and severe depression up to 2 years before diagnosis along with rapid cognitive decline rapidly starting 3 months prior to admission	Moderate oppositionality and agitation, Echolalia, palilalia, paratonia, perioral involuntary movements, end – point tremor, brisk tendon reflexes, presence of masseter reflex and primitive reflexes, atypical gait disturbances	Yes	White matter hyperintensities (Fazekas 1) Sheltens 1	No	Presence of sharp waves with left predominance	Normal

7/F/21	Autoimmune encephalitis/ extracellular antigen	NMDAR	No	Yes	No	Emotional and cognitive disorder including memory and concentration deficits along with perceptual disturbances, impaired speech and presence of four epileptic seizures 3 months prior to admission	Confusion, disorientation, irritability – oppositionality, emotional lability, echolalia & palilalia, irritability, presence of nystagmus, brisk tendon reflexes, paroxysmal, oral & limb dyskinesias, impaired speech	Yes	Normal	No	Focal slow delta wave in the left hemisphere and sporadically sharp wave in both hemispheres (mainly left)	Normal
8/F/20	Autoimmune encephalitis/ extracellular antigen	NMDAR	No	Yes	No data	Acute behavioral changes, disorientation and epileptic seizures including focal tonic seizures with secondary generalisation starting two months prior to admission	Confusion, disorientation, personality changes, hypertonia, mild cerebellar disorder, brisk tendon reflexes	Mild	Normal	Yes; Ovarian tumour	Generalized slowing, epileptiform discharges	No data
9/F/71	Autoimmune encephalitis/ extracellular antigen	GABA1R	Yes	Yes	No	Episodic short-term memory impairment, confusion and disorientation of place and person 15days prior to admission of subacute onset	Irritability, oppositionality, agitation, emotional lability, cognitive decline – memory deficits	Yes	White matter hyperintensities (Fazekas 1)	No	Normal	No data

						along with four epileptic seizures a few days before hospitalisation (eyes fixation to the right, jerky movements of the right arm and shoulder and hypersalivation for 20 - 30 mins, that were followed by postictal confusion)						
10/M/42	Autoimmune encephalitis/ extracellular antigen	GlyR	Yes	No	Yes; Ankylosing spondylitis (axonal type)	Symptoms of fatigue and physical weariness even during minimal activity (activities of daily life etc) with concomitant palpitations, muscle cramps and left eyelid ptosis (blepharospasm) along with gait disturbances and walking impairment ("rigid walking") as well as startle - response to auditory or emotional stimuli 3 years prior to diagnosis	Asymmetric eyelid ptosis – blepharospasm, upper and lower limb hypertonia and paratonia (occasional finding), action tremor (only during slow movements), gait ataxia, brisk tendon reflexes with presence of clonus, acquired hyperekplexia (at auditory	No	Normal	No	No data	Normal

							stimuli), signs of autonomic dysfunction (tachyarrythmia, mild urinary incontinence, blood pressure abnormalities), sleep apnea,					
11/F/41	Autoimmune encephalitis/ synaptic antigens	GAD	Yes	Yes	Yes; Vitiligo	History of left mallet 2 years prior to diagnosis along with dysesthesias and brief episodes of sudden stiffness of the left leg, which used to awake the patient. Symptoms aggravated 6months prior to diagnosis.<br>Note that this patient had history of epilepsy with complex focal seizures since the age of 26.	Parkinsonism with left lower extremity hypertonia – rigidity (distally > proximally), paroxysmal dyskinesias and dystonia, brisk tendon reflexes with left exaggeration	No	Neuroglial cyst/ dilated perivascular space in the left insular area	No	Normal	No data
12/M/76	Paraneoplastic encephalitis/ intracellular antigens	Ma2/Ta	Yes	No	No	Symptoms of instability followed by falls and memory disorder in a	Cognitive decline – memory deficits parkinsonis	Yes	Diffuse cerebral and cerebellar atrophy,	Yes; Cancer of nasopharynx	Normal	Normal

						patient with a past medical history of nasopharyngeal Ca diagnosed 4 years prior to neurological disease and treated with chemotherapy and radiotherapy.	m with rigidity – hypertonia bradykinesia and impaired postural reflexes, gait and limb ataxia, brisk tendon reflexes, autonomic nervous system manifestatio ns – orthostatic hypotension		diffuse small vessel disease			
13/M/71	Paraneoplast ic encephalitis/ intracellular antigens	CRMP-5/CV2	Yes	No	No data	Cognitive impairment with visuospatial skill deficits, psychotic symptoms, peripheral neuropathy and parkinsonism developed gradually during the last 3 years before diagnosis	Cognitive deficits with spatial disorientatio n, memory and concentratio n impairment, irritability, oppositionali ty, emotional lability, personality changes, thought disorder – psychosis, hypertonia, reduced superficial	Yes	White matter hyperintensitie s (Fazekas1) Sheltens 1	Yes; Possibl e lung cancer	Normal	Sensory – motor neuropat hy

							and proprioceptive sensation,					
14/F/69	Paraneoplastic encephalitis/ intracellular antigens	CRMP-5/CV2, Yo	Yes	No	No	Gait instability leading to falls that followed a slow progressively deterioration along with urgent urination and mild memory disorder starting 2 years prior to diagnosis in a patient with a history of lung cancer diagnosed 7 years before hospitalisation. Severe discomfort in the lower/upper extremities and torso was experienced by the patient. Sudden deterioration of gait instability presented up to 6 months before hospitalisation causing the patient to be bedridden 1 month before admission	Cognitive decline with memory and concentration deficits, agitation, emotional lability – depression, dysarthria, nystagmus, hypertonia – paratonia, bradykinesia, limb ataxia, severe gait disturbance (inability to stand or walk), brisk tendon reflexes, reduced superficial and proprioceptive sensation, urinary incontinence	Yes	White matter hyperintensities (Fazekas 2), predominantly increased signal in the left basal ganglia on T2-weighted and FLAIR images	Yes; Non-small cell lung cancer	Normal	Sensory axonal neuropathy
15/F/85	Paraneoplastic encephalitis/	Yo	Yes	No	No	Gait and motor impairment (instability and	Cognitive decline, confusion –	Yes	White matter hyperintensities (Fazekas 2),	Yes;	No data	Normal

	intracellular antigens					weakness of the lower extremities) rapidly deteriorating up to 2.5 months before admission in a patient with a two-year history of parkinsonism moderately responding to treatment	disorientation, irritability, oppositionality, emotional lability, psychosis – delirium, echolalia, personality changes, swallowing difficulty, involuntary movements of the eyes resembling opsoclonus, mixed type of tremor of the upper and lower extremities, hypertonia – paratonia, bradykinesia, limb and sever gait ataxia (bedridden), sleep disturbance, weight loss		diffuse atrophy (including temporal horns and hippocampal areas) - widening of the ventricles disproportionate to the enlargement of the peripheral subarachnoid spaces of the brain	Endometrial cancer		
16/M/72	Paraneoplastic encephalitis/ intracellular antigens	Yo	Yes	No	No	Weakness, involuntary weight loss, tremor of the upper extremities and dry mouth,	End – point intention tremor, gait and limb ataxia, decreased	No	Normal Fazekas 1	Yes; Possible prostate cancer	Normal	Sensory axonal neuropathy

						following viral respiratory infection 1 month before admission	proprioception in lower extremities, weight loss					
17/F/73	Paraneoplastic encephalitis/ intracellular antigens	Zic-4	Yes	Yes	History of allergic reactions	Instability and short memory deficits and executive dysfunction 5 to 6 months prior to admission	Cognitive decline – memory & concentration deficits, spatial disorientation, confusion – disorientation, irritability, hyperthymia, emotional lability, personality change, nystagmus, mild hypotonia, stereotypical movements of the back and shoulders during speech, mild bradykinesia, end – point tremor, gait and limb ataxia, pyramidal signs, weight	Yes	White matter hyperintensities (Fazekas 2)	Yes; Possible mediastinal tumour	Slow activity mainly in the right temporal areas and FIRDA	Normal

							loss, sleep disturbances					
18/F/82	Paraneoplastic encephalitis/ intracellular antigens	CRMP-5/CV2 Hu	Yes	No	No	Deterioration of her motor ability (severe instability, frequent falls), dysarthria and upper limbs dysfunction starting 5 months before admission and gradually developing leading to the patient being bedridden with severe dysarthria (incomprehensible speech)	Severe dysarthria, nystagmus, dysmetric saccades, difficulty in swallowing, mild to moderate limb and severe gait ataxia (inability to stand or walk), pyramidal signs	Not prominent	Diffuse cortical and cerebellar atrophy	Yes; Possible lung cancer	Normal	Sensory axonal neuropathy
19/M/63	Paraneoplastic encephalitis/ intracellular antigens	Amphiphysin	Yes	No	No data	Symptoms of gait instability and dysarthria along with oscillations 1 year before admission to the hospital	Nystagmus, parkinsonism with hypertonia, gait and limb ataxia, pyramidal signs	No	Normal		Normal	No data
20/M/72	Paraneoplastic encephalitis/ intracellular antigens	CRMP-5/CV2 GAD (low titer)	Yes	No data	No	Confusion and disorientation in time - space, impairment in cognition - mainly memory - as well as lower performance in Activities of Daily Living, at least 10 days prior to admission.	Cognitive decline – spatial disorientation, short-term memory deficits (inability to register new information), confusion, occasional	Yes	High signal intensity in T2 in the hippocampi and amygdaloid nuclei bilaterally Frontal atrophy	Yes; Lung cancer, adrenal gland metastasis	Theta and delta waves bifrontal and bitemporal areas	Severe sensorimotor axonal neuropathy

						Worsening of symptoms reported a few days before hospitalization.	oppositionality and irritability, mild end-point intention tremor					
21/F/58	Autoimmune encephalitis/ synaptic antigens	GAD	Yes	Yes	Yes; Hashimoto's thyroiditis	Psychiatric manifestations including symptoms of anxiety, phobias and depression, behavioural disorders, gradual cognitive dysfunction as well as visual disturbances (occasional oscillopsia) starting about 3 years before diagnosis. Patient also developed Diabetes mellitus type I with episodes of metabolic acidosis and other systemic complications (femoral vein thrombosis). Motor impairment, including gait problems for the last 1.5 years	Global cognitive decline, confusion, disorientation, irritability, agitation, oppositionality, personality changes, emotional lability, echolalia, palilalia, nystagmus, macro – square wave jerks, ocular flutter, diffuse muscle atrophy, mixed limb and axonal hypertonia - rigidity and paratonia, diffuse bradykinesia, impaired	Yes	Cortical atrophy and dilatation of the ventricular system over time	No	Moderate organization, no stable basic rhythm, interchanged with slow and fast rhythms	Focal denervation

						prior to diagnosis. Symptoms followed marked deterioration with patient showing psychomotor agitation, cognitive impairment, frequent falls - inability to walk independently as well as complete functional decline before admission.	postural reflexes, brisk tendon reflexes, weight loss					

Abbreviations: NMDAR= N-methyl D-aspartate receptors; CRMP-5/ CV2= collapsin response mediator protein -5 / CV2; Ma2 = membrane active protein 2; CASPR2 = contactin-associated protein-like 2; FLAIR = fluid- attenuated inversion recovery; GABABR = gamma-aminobutyric acid receptor B; Zic4 = Zic family member 4; GAD = glutamic acid decarboxylase; GlyR = glycine receptor, LGI1 = leucine-rich, glioma inactivated 1; mGluR = metabotropic glutamate receptor; EEG = electroencephalogram; MRI = magnetic resonance imaging; CSF = Cerebrospinal fluid; EMG = electromyography

Supplementary Table 2: Significant differences in various parameters tested among patients with autoimmune encephalitis with autoantibodies against extracellular versus intracellular antigens (without correction for multiple comparisons).

**Autoimmune encephalitis (AE) with autoantibodies against extracellular VS intracellular / synaptic antigens**			
	P value (Mann-Whitney test, Alpha p value threshold; 0,05)	Mean rank of AE with Extracellular Antigen	Mean rank of AE with Intracellular Antigen
T-Tau protein	0,000108	6,000	15,55
Presence of dysdiadochokinesia	0,003870	7,500	14,18
Presence of signs of parkinsonism (bradykenia, tremor, rigidy) (at least one)	0,003870	7,500	14,18
Plasmapheresis as treatment modality performed during acute treatment	0,003870	14,30	8,000
NFL (pg/mL)	0,004309	7,100	14,55
Age at disease onset (years)	0,005106	7,150	14,50
Death as outcome during follow up	0,008242	6,500	11,83
Presence of balance disorder	0,008922	7,600	14,09
Median hospitalization time period (in days)	0,022666	12,33	6,667
Immunoglobulin IgG serum levels at disease onset	0,028127	10,38	5,286
Tumor presence during follow up	0,029973	8,100	13,64
NMDAR encephalitis as final diagnosis	0,035088	13,20	9,000
Presence of bradykinesia	0,035088	8,500	13,27
Presence of peripheral neuropathy	0,035088	8,500	13,27

Abbreviations: T-Tau protein; Total tau protein, NFL; neurofilaments, NMDAR; N-methyl D-aspartate receptors.

Supplementary Table 3: Significant differences in various parameters tested among patients with autoimmune encephalitis associated or not with the presence of an underlying tumor (without correction for multiple comparisons).

**Autoimmune encephalitis (AE) associated with the presence of an underlying tumor or not.**			
	P value (Mann-Whitney test, Alpha p value threshold; 0,05)	Mean rank of AE not associated with tumor.	Mean rank of AE associated with tumor.
NFL (pg/mL)	0,000717	6,909	15,50
Presence of cerebellar ataxia	0,001905	7,455	14,90
Death as outcome during follow up	0,008242	6,500	11,83
Age at disease onset (years)	0,016719	7,955	14,35
Presence of dysdiadochokinesia	0,023736	8,455	13,80
Presence of dysmetria	0,023736	8,455	13,80
Tandem gait difficulty	0,029973	8,409	13,85
Presence of balance disorder	0,029973	8,364	13,90
CRMP-5/ CV2 associated encephalitis as final diagnosis	0,035088	9,000	13,20
Stiff person spectrum disorders as final diagnosis	0,035088	13,27	8,500
T-tau protein	0,035700	8,273	14,00
Immunoglobulin IgG serum levels at disease onset	0,047752	9,889	5,167

Abbreviations: T-Tau protein; Total tau protein, NFL; neurofilaments, NMDAR; N-methyl D-aspartate receptors, CRMP-5/ CV2; collapsin response mediator protein -5 / CV2.
